# In-vitro and in-vivo antioxidant assays of chicory plants (*Cichorium intybus* L.) as influenced by organic and conventional fertilisers

**DOI:** 10.1186/s12870-020-2256-2

**Published:** 2020-01-20

**Authors:** Lovro Sinkovič, Polona Jamnik, Mojca Korošec, Rajko Vidrih, Vladimir Meglič

**Affiliations:** 1 Crop Science Department, Agricultural Institute of Slovenia, Hacquetova ulica 17, SI-1000 Ljubljana, Slovenia; 2 Department of Food Science and Technology, Biotechnical Faculty, University of Ljubljana, Jamnikarjeva 101, SI-1000 Ljubljana, Slovenia

**Keywords:** Antioxidant potential, *Cichorium intybus*, Fertilisers, Phenolics content, *Saccharomyces cerevisiae*

## Abstract

**Background:**

Chicory (*Cichorium intybus* L.) is a traditional European crop that is highly appreciated for its contents of bioactive compounds, especially phenolics, which have high antioxidant activities. Among other factors, agricultural practice might affect the contents of these bioactive compounds, which are also important from a nutritional point of view, and affect the shelf-life.

**Results:**

The antioxidant potential (AOP) of chicory plants treated with different fertilisers was investigated in vitro using DPPH radical scavenging and in vivo using the yeast *Saccharomyces cerevisiae*. Additionally, total phenolics content (TPC) was evaluated using Folin–Ciocalteu reagent, and total flavonoids content (TFC) using the aluminium chloride method. Four different chicory cultivars were included: ‘Treviso’, ‘Verona’ and ‘Anivip’ as red cultivars; and ‘Castelfranco’ as a red-spotted cultivar. These were grown in pots under controlled glasshouse conditions using organic and/or mineral fertilisers. The combination of organic and mineral fertilisers during red chicory growth resulted in significantly higher in-vitro and in-vivo AOPs compared to the control. For the red-spotted cultivar ‘Castelfranco’, this combined organic and mineral fertilisation decreased AOPs in vitro and increased AOPs in vivo. Among the cultivars examined, ‘Castelfranco’ treated with combined organic plus mineral fertilisers showed the highest AOP in vivo, accompanied by the lowest TPC and TFC.

**Conclusions:**

These data show that application of different fertilisers has different impacts on red and red-spotted chicory cultivars in terms of TFC and TPC, which for red-spotted chicory resulted in different AOPs in vitro and in vivo. The in-vitro AOP is well reflected in the in-vivo AOP for the red chicory cultivars, but less so for the red-spotted cultivar ‘Castelfranco’. Based on the in-vivo AOPs for these chicory cultivars analysed, the combined organic plus mineral fertiliser treatment is recommended.

## Background

*Cichorium intybus* L. is commonly known as chicory. It belongs to the family Asteraceae, and is widely distributed around the world, according to various uses. Chicory plants can be cultivated for food as the leaves, rosettes and heads, which are usually eaten raw in salads. Chicory has become an important vegetable and technical crop in many temperate regions over the last decade, especially in Europe, Asia and North America [1].

One of the core ideas behind organic production compared to conventional production is that the cropping system should be less dependent on the import of resources, and its negative effects on the surrounding environment should be minimised [2]. Organic farming using organic fertilisers and cover crops instead of mineral fertilisers is growing rapidly, particularly with bans on the use of various pesticides, herbicides, hormones and other chemicals [3]. Organic farming also has the potential for production of healthier food, and it has been adopted for a wide range of climate and soil types. Furthermore, the perception among consumers is that organically produced crops have greater nutritional value, although this is affected by many factors [4]. However, in the practical world of farming, there is a tendency for organic production methods to become more like conventional methods, with increased reliance on input factors.

A number of comparative studies have shown higher levels of phenolic compounds (phenolics) in organic plant products [5–8], although the variations across these studies have been wide, as these levels depend on plant fertilisation, ripening stage, plant age at harvest, and weather conditions [9].

Phenolics are secondary metabolites that have physiological and morphological importance in plants, including chicory. As well as contributing towards the colour and sensory characteristics of vegetables, phenolics have important roles in plant growth and reproduction [10]. Chicory is a rich source of bioactive compounds, such as tannins, saponins and flavonoids [10]. Among the flavonoids, Innocenti et al. [11] reported cyanidin 3-O-glucoside, delphinidin 3-O-(6′′ malonyl)-glucoside and cyanidin 3-O-(6′′ malonyl)-glucoside for the chicory cultivar ‘Treviso’. Carazzone et al. [12] investigated the chicory cultivars ‘Chioggia’, ‘Treviso’, ‘Treviso tardivo’ and ‘Verona’, and they reported the flavone derivatives apigenin-7-O-glucoside for ‘Chioggia’ and ‘Verona’, and chrysoeriol-3-O-glucoside for ‘Chioggia’. In terms of anthocyanidin derivatives, Carazzone et al. [12] confirmed cyanidin-3-O-glucoside, cyanidin-3-O-galactoside and cyanidin-3-O-(6″-O-acetyl)-glucoside for all of these cultivars, while cyanidin-3-O-(6″-O-malonyl)-glucoside and petunidin-3-O-(6″-O-malonyl)-glucoside were found only in ‘Chioggia’. They also reported cyanidin-3,5-di-O-(6″-O-malonyl)-glucoside for all of these cultivars, delphinidin 3-O-(6″-O-malonyl)-glucoside-5-O-glucoside and pelargonidin-3-O-glucuronide for all cultivars except ‘Verona’, and malvidin-3-O-glucoside for cultivar ‘Verona’ only.

Anthocyanidins have been reported to reduce intracellular reactive oxygen species [13]. In our previous studies, we showed that the phenolic and fatty-acids profiles of chicory are highly influenced by both the cultivar and the fertilisers used [14, 15]. Phenolics are also considered as important functional food components, and together with other compounds, they represent a wide range of natural substances in leafy vegetables that are beneficial to human health, including in chicory [16, 17]. Consumption of fresh vegetables, and therefore dietary compounds such as antioxidants, has many health benefits, especially for the promotion of lower prevalence of cardiovascular diseases and cancers, and protection against neurological decline [18, 19].

A rich area of research has been developed to investigate the effects of bioactive plant components in foods that are not related to direct antioxidant actions. These bioactive plant components should thus be considered simply in terms of their chemical properties, and not automatically related to any equivalent function in vivo. Therefore, it is essential to determine the antioxidant effects of such bioactive plant components at multiple levels, both in vitro and in vivo, to obtain the full picture of their activities, which might be relevant to various physiological and pathological states in humans [20].

There is strong epidemiological evidence that antioxidants derived from fruit and vegetables can protect the body against various diseases. Chicory is a good source of phenolics [14, 15] that can scavenge free radicals both in vitro and in vivo. Phenolics might also act in vivo through the expression/ repression of genes. The in-vitro antioxidant capacity of some plants is significantly correlated to their total phenolics content (TPC), while this is only an approximate reflection of the in-vivo antioxidant potential (AOP) in other plants [21, 22]. These differences might be due to the solubility, bioavailability and/or metabolism of these antioxidant phenolics. Wang et al. [23] demonstrated that extracts of lychee fruit pericarp can have anticancer activities against hepatocellular carcinoma. In particular, the lychee fruit pericarp contains condensed tannins (polymeric proanthocyanins), epicatechin, procyanindin A2 and flavonoids [23].

To the best of our knowledge, there have not been any studies on the in-vivo antioxidant activities of chicory cultivars produced using different fertilisers (i.e., organic, mineral, combination of organic and mineral). The aim of the present study was thus to determine the in-vitro and in-vivo AOPs of four chicory cultivars to define how they are influenced by commercially available organic and/or mineral fertilisers. Further, we wanted to find out how the in-vitro AOP of chicory correlates to the in-vivo AOP, according to cultivar and fertiliser type.

## Results

### In-vitro *and* in-vivo *antioxidant activities*

**Fig. 1:**
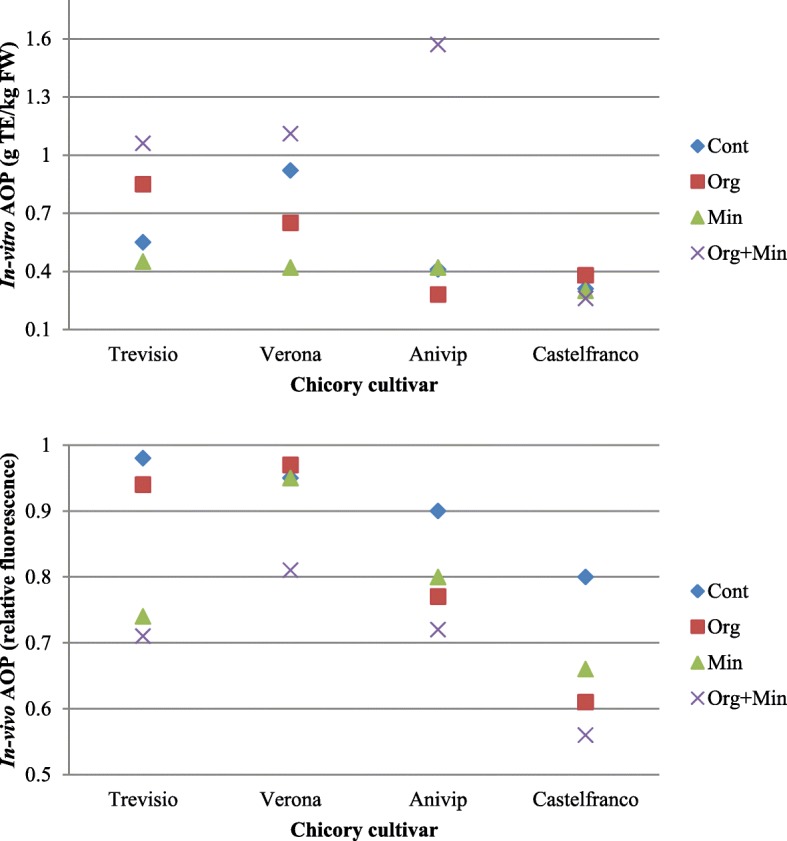
Scatter plots for the influence of the different fertiliser treatments on the in-vitro (top) and in-vivo (bottom) antioxidant potential (AOP) for the four chicory cultivars. TE, Trolox equivalents; FW, fresh weight; Cont, Control; Min, Mineral; Org, Organic; Org + Min, Organic+Mineral

The influence of the fertiliser treatments on in-vitro and in-vivo AOP for the four chicory cultivars is shown in Fig. 1 and Additional file 1: Table S1. Compared to the Control, the highest in-vitro AOP was seen for the red cultivars ‘Treviso’, ‘Verona’ and ‘Anivip’ when treated with Organic+Mineral fertiliser (1.06, 1.11, 1.57 g TE/kg FW, respectively). Treatment of the red cultivars with other fertilisers (Organic or Mineral fertilisers) decreased the in-vitro AOP (except for cultivar ‘Treviso’ for Organic, ‘Anivip’ for Mineral fertiliser). For cultivar ‘Castelfranco’, the highest in-vitro AOP compared to the Control was for Organic fertiliser treatment (0.38 g TE/kg FW). Treatment of ‘Castelfranco’ with Organic+Mineral fertiliser resulted in lower AOP compared to the Control (0.26 g TE/kg FW), or the same compared to the Control for the Mineral fertiliser. For the in-vivo AOP for all of the chicory cultivars, this was highest compared to the Control for Organic+Mineral fertiliser treatment (0.71, 0.81, 0.72, 0.56, respectively). For the other fertilisers, compared to the Control, the in-vivo AOP data were either higher or the same (cultivar ‘Treviso’ for Organic, ‘Verona’ for Organic or Mineral fertiliser).

**Fig. 2:**
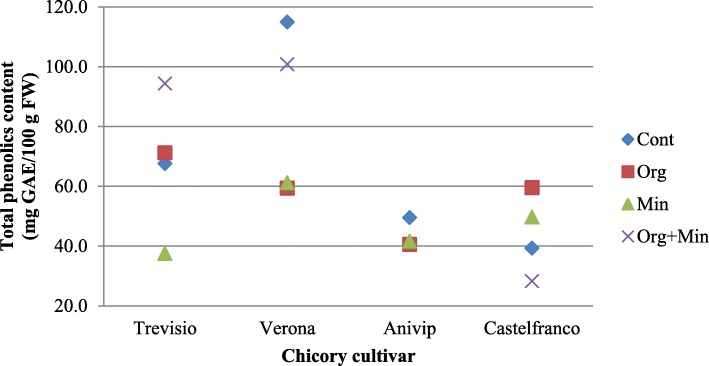
Scatter plot for the influence of the different fertiliser treatments on the total phenolics content for the four chicory cultivars. GAE, gallic acid equivalents; FW, fresh weight; Cont, Control; Min, Mineral; Org, Organic; Org + Min, Organic+Mineral

**Fig. 3:**
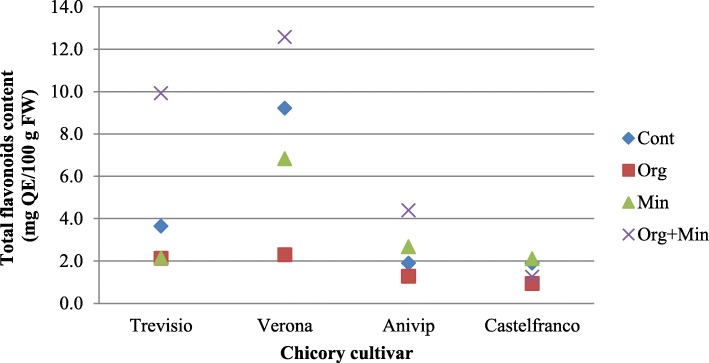
Scatter plot for the influence of the different fertiliser treatments on the total flavonoids content for the four chicory cultivars. QE, quercetin equivalents; FW, fresh weight; Cont, Control; Min, Mineral; Org, Organic; Org + Min, Organic+Mineral

For all four of the chicory cultivars treated with the different fertilisers, TPC and TFC were measured (Figs. 2 and 3; Additional file 2:Table S2 and Additional file 3:Table S3). In the red cultivars ‘Trevisio’, ‘Verona’ and ‘Anivip’ treated with Organic+Mineral fertiliser, compared to the Control there was higher TPC (except for cultivar ‘Verona’) (94.33, 100.80, 120.20 mg GAE/100 g FW, respectively) and TFC (9.92, 12.58, 4.39 mg QE/100 g FW, respectively). Treatment of red cultivars with only Organic or only Mineral fertilisers decreased TPC (except for cultivar ‘Treviso’) and TFC (except for cultivar ‘Anivip’). Cultivar ‘Castelfranco’ treated with Organic or Mineral fertiliser had higher TPC, with lower or the same TFC, compared to the Control, respectively. Treatment of ‘Castelfranco’ with Organic+Mineral fertiliser decreased TPC (28.27 mg GAE/100 g FW) and TFC (1.25 mg QE/100 g FW), compared to the Control.

### Correlations between the study variables

Table 1 shows the data for the Pearson’s correlation coefficients among the studied variables of in-vitro AOP, in-vivo AOP, TPC and TFC for all four of the chicory cultivars grown under the different fertiliser treatments. Significant correlations between in-vitro AOP and TPC (0.916***), in-vitro AOP and TFC (0.621***), and TPC and TFC (0.735***) were found.

**Table 1:** Pearson’s correlation coefficients among the variables determined for the four chicory cultivars grown under the different fertiliser applications

Variable	Variable			
	1	2	3	4
	In-vivo antioxidant potential	In-vitro antioxidant potential	Total phenolics content	Total flavonoids content
In-vivo antioxidant potential		ns	ns	ns
In-vitro antioxidant potential	0.128		***	***
Total phenolics content	0.226	0.916		***
Total flavonoids content	0.206	0.621	0.735	

Significance of the correlations: ***, *P* < 0.001; ns, not significant

The Pearson’s correlation coefficients between in-vivo and in-vitro AOP, TPC and TFC for the individual chicory cultivars are reported in Table 2. Negative correlations between in-vivo AOP and in-vitro AOP (− 0.713**), and in-vitro AOP and TFC (− 0.803**) were found for the ‘Verona’ cultivar, and between in-vivo AOP and in-vitro AOP (− 0.582*) for the ‘Anivip’ cultivar. Furthermore, positive correlation between in-vivo AOP and TFC (0.637*) was found for the red-spotted cultivar ‘Castelfranco’.

**Table 2:** Pearson’s correlation coefficients between in-vivo AOP and in-vitro AOP, TPC, and TFC for individual chicory cultivars regardless of fertiliser treatments

Correlant	Correlation coefficient (R^2^) for in-vivo antioxidant potential according to cultivar			
	‘Trevisio’	‘Verona’	‘Anivip’	‘Castelfranco’
vs. in-vitro AOP	−0.255	−0.713	− 0.582	0.086
*P-*value	ns	**	*	ns
vs. TPC	−0.022	− 0.443	−0.540	0.033
*P-*value	ns	ns	ns	ns
vs. TFC	−0.534	−0.803	− 0.570	0.637
*P*-value	ns	**	ns	*

*AOP*, antioxidant potential, *TPC* total phenolics content, *TFC* total flavonoids content

Significances of the correlations: *, *P* < 0.05; **, *P* < 0.01; ns, not significant

### Multivariate analysis

**Fig. 4:**
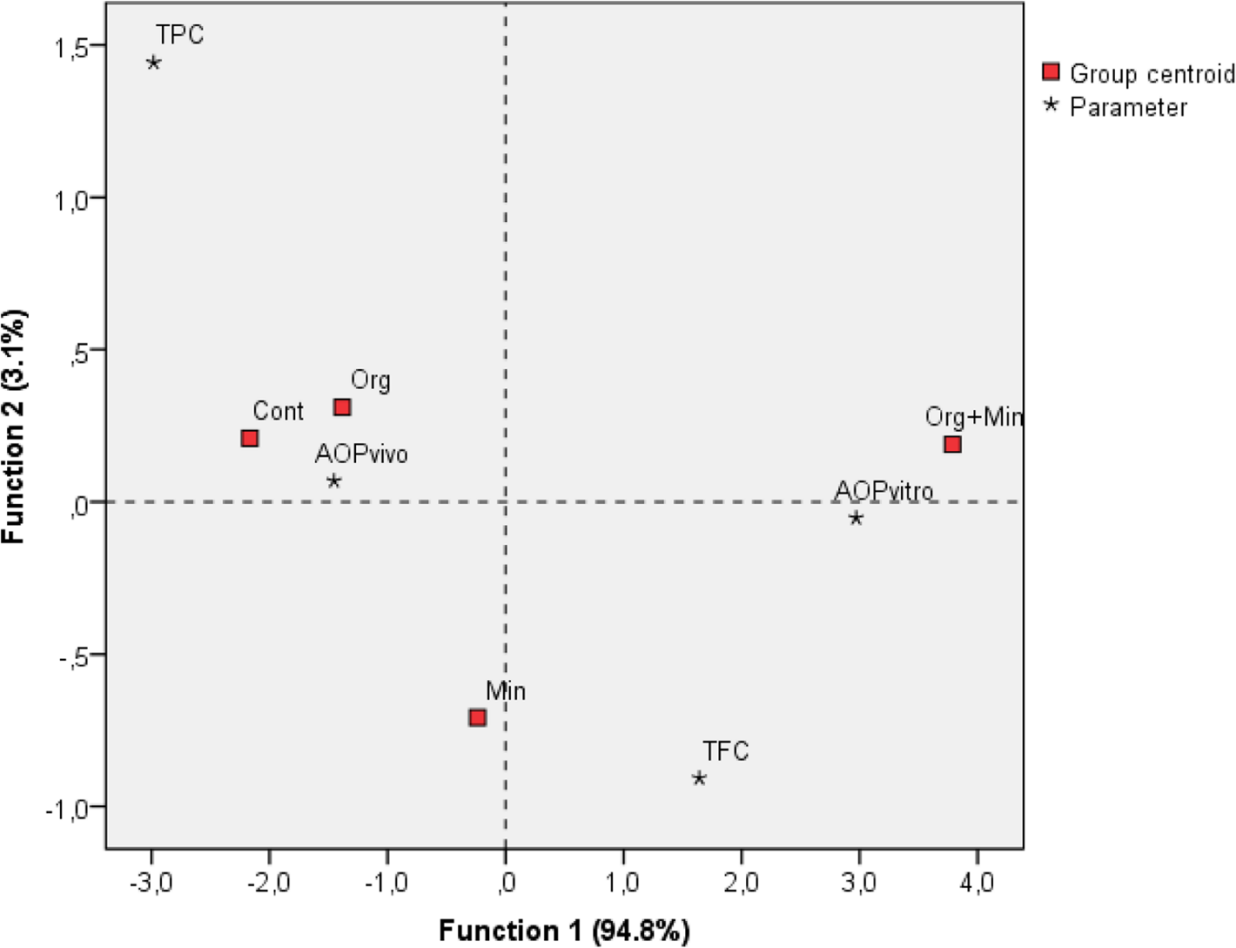
Biplot for the linear discriminant analysis from the data (see text for details) for the samples from the four chicory cultivars grown under the four fertiliser treatments. Cont, Control; Min, Mineral; Org, Organic, Org + Min, Organic+Mineral; AOPvivo, in-vivo antioxidant potential; AOPvitro, in-vitro antioxidant potential; TPC, total phenolics content; TFC, total flavonoids content

A biplot was constructed for the first two functions, which shows how the treated cultivars are different and which parameter is mainly responsible for the variation. The discrimination across the four variables determined in the chicory samples for these three different fertiliser treatments and the Control is shown in Fig. 4. The first two dimensions of the data account for 97.9% of the information contained in the data. The Group centroids show clear discrimination among the groups of chicory samples for the three fertiliser treatments and the Control (Fig. 4). The model enables 87.5% correct classification of originally grouped cases, where 100.0% correct classification is predicted for samples of chicory for the combination of the organic and mineral fertilisers, and for samples where the organic fertiliser was used. For the Control group and the mineral fertiliser group, 75.0% correct classification was achieved.

## Discussion

### In-vitro *and* in-vivo *antioxidant activities*

Vegetables, including chicory, are rich external sources of dietary antioxidants that are important for the human body [24]. Studies to date have shown that in-vitro AOP and TPC in vegetables grown under organic and conventional production practices can be influenced by the practice followed [3, 7, 9]. The aim of the present study was to investigate the effects of the application of different fertiliser treatments (i.e., Organic, Mineral, Organic+Mineral) compared to the Control on in-vitro and in-vivo AOP, and on TPC and TFC for four different chicory cultivars. Based on previous studies [14, 15, 25, 26], the chicory cultivars included in the present study have high in-vitro AOP due to high TPC.

For the red cultivars ‘Treviso’, ‘Verona’ and ‘Anivip’ compared to the Control, the in-vitro AOPs were highest for the Organic+Mineral fertiliser treatment. The exception was the red-spotted cultivar ‘Castelfranco’, which showed highest in-vitro AOP for Organic fertiliser treatment, while the Organic+Mineral fertiliser treatment resulted in the lowest AOP among these fertiliser treatments. Treatment of the red cultivars with other fertilisers (Organic or Mineral fertiliser) mostly decreased in-vitro AOPs, with the exception of cultivars ‘Treviso’ and ‘Anivip’. Application of different fertilisers for cultivar ‘Castelfranco’ resulted in different effects that depended on the fertiliser type (Fig. 1, Additional file 1: Table S1).

However, the AOP of bioactive compounds in the cell cannot be predicted merely on the basis of in-vitro studies of AOPs of such extracts [27]. Therefore, the effects of the extracts from these differentially treated chicory cultivars on the AOP was investigated in the cells, using the yeast *S. cerevisiae* as the model organism. These data for the in-vivo AOPs are expressed as the relative fluorescence of the *S. cerevisiae*, whereby lower values indicated higher AOPs. Similar to the in-vitro studies, for the red cultivars of ‘Trevisio’, ‘Verona’ and ‘Anivip’, compared to the Control, the highest AOP was with the Organic+Mineral fertiliser treatment. However, in contrast to the in-vitro study for the red-spotted cultivar ‘Castelfranco’, this also showed the highest in-vivo AOP when treated with the Organic+Mineral fertiliser, which was thus similar to the red cultivars here.

These data indicate that compared to the Control, the application of these different fertiliser treatments significantly influenced the AOPs both in-vitro and in-vivo. Furthermore, the high similarity between the in-vitro and in-vivo AOPs seen for the red chicory cultivars with the Organic+Mineral fertiliser showed this to be the most suitable treatment for these cultivars. On the other hand, compared to the Control, the red-spotted cultivar ‘Castelfranco’ showed highest in-vivo AOP among the fertiliser treatments for Organic+Mineral, where the in-vitro AOP was lowest (Fig. 1). Interestingly, the in-vivo AOPs for all of the cultivars and fertilisers used were either higher or showed no effects compared to the Control regardless of the different effects obtained in the in-vitro studies. For all of the chicory cultivars the highest in-vivo AOP compared to the Control was detected for Organic+Mineral fertiliser treatment. The comparison of the red and red-spotted cultivars with the Organic+Mineral fertiliser treatment showed that ‘Castelfranco’ had the highest in-vivo AOP (Fig. 1, Additional file 1: Table S1).

Thus, measurements of the in-vitro AOP of extracts/ compounds can be used as a first screening to help to define them as extracts/ compounds with potential antioxidant activity before the use of in-vivo models. However, while in-vitro tests are used to demonstrate the intrinsic activities of the compounds, in-vivo assays are focused on the physiological effects, to provide a second, and essential, line of evidence for antioxidant activity [28, 29].

To better explain these similarities and differences between the in-vitro and in-vivo AOP data, TPC and TFC were also evaluated (Figs. 2 and 3; Additional file 2: Table S2 Additional file 3: Table S3). The reasoning followed was that the application of different fertilisers can influence the general chemical composition of chicory plants, which will including their production of secondary metabolites, such as TPCs and TFCs, as stress responses [14, 15, 28], which will then result in different antioxidative activities. For the red cultivars, compared to the Control, Organic+Mineral fertiliser treatment resulted in higher TPCs (except for cultivar ‘Verona’) and TFCs. Only Organic or only Mineral fertiliser treatment decreased TPCs (except for cultivar ‘Treviso’) and TFCs (except for cultivar ‘Anivip’). On the other hand, treatment of the red-spotted cultivar ‘Castelfranco’ with Organic+Mineral fertiliser resulted in decreased TPC and TFC. Cultivar ‘Castelfranco’ treated with Organic or Mineral fertiliser had higher TPC, with lower or the same TFC compared to the Control, respectively. Indeed, across all of the cultivars examined under each of the fertiliser treatments, TFCs were lowest for ‘Castelfranco’. Additionally, ‘Castelfranco’ had the lowest TPCs for Organic and Mineral fertiliser treatments, where the highest in-vivo AOP compared to the Control was detected.

Additionally, in our previous study we showed that compared to the red cultivars, ‘Castelfranco’ treated with Organic+Mineral fertiliser had considerably lower levels of hydroxycinnamic acids, flavonols and other unknown phenolic compounds [14, 15]. D’Evoli et al. [26] compared ‘Treviso’ and ‘Castelfranco’ chicory plants, and showed that anthocyanin contents are much higher for ‘Treviso’ compared to ‘Castelfranco’ (13–45 vs. 4–6 mg/100 g FW, respectively). Additionally, as the main anthocyanin in red and red-spotted chicory [11, 30], cyanidin-3-O-(6”malonyl)-glucoside contributed to about 8% of the total anthocyanins in ‘Treviso’ and 16% in ‘Castelfranco’. Cyanidin 3-*O*-glucoside has shown high, similar, and even higher in-vivo AOP compared to tocopherols [31]. Similarly, Slatnar et al. [27] showed differences in antioxidant activities of berry juices between in-vitro and in-vivo studies. Here, the uptake of phenolic compounds into the cells was shown to be an important factor. In addition to phenolic compounds entering the cells, a key factor in the determination of in-vivo antioxidative activity of berry juices was the ratio between the particular phenolic compounds. Where there were high anthocyanins content and very low flavonol and hydroxycinnamic acid contents, lower intracellular oxidation was detected. Specifically, AOP in-vivo decreased with higher consumption of hydroxycinnamic acids and lower consumption of anthocyanins in the cells of *S. cerevisiae.* Thus, rather than TPC and/or TFC themselves, the main reason for the differences between in-vivo and in-vitro AOP studies might be more directly related to the uptake of, and specifically to the ratio between, the particular compounds that enter the cells. Namely, the antioxidative activity of compounds depends on their uptake into cells, where the balance between their lipophilicity and hydrophilicity has important role [32]. Then in the cell the reduction potential and antioxidant defence systems have additional effect on their antioxidative activity. Furthermore, different mechanisms of antioxidative activities might be expressed at different concentrations of the compounds tested. The antioxidants can act as scavengers of free radicals or indirectly by inhibition of the activity or expression of enzymes generating free radicals or by induction of the activity or expression of cell endogenous antioxidant defence systems [33].

### Correlations between the study variables

In the comparisons of the means of the different cultivars and treatments, there were highly significant correlations (*P* < 0.001) between in-vitro AOP and TPC, in-vitro AOP and TFC, and TPC and TFC (Table 1). As lower values for in-vivo AOP mean higher in-vitro AOP, negative correlations between these two parameters were expected, as well as between in-vivo AOP and TPC, and in-vivo AOP and TFC.

Relations between in-vivo and in-vitro AOP, TPC and TFC for cultivars ‘Verona’, ‘Anivip’ and ‘Trevisio’, respectively, tend to be negative, while they are positive for ‘Castelfranco’ (Table 2). Strong negative correlations between in-vivo AOP and in-vitro AOP, and in-vitro AOP and TFC were confirmed for the ‘Verona’ cultivar, and moderate negative correlation between in-vivo AOP and in-vitro AOP for the ‘Anivip’ cultivar. For the red-spotted cultivar ‘Castelfranco’, there was significant positive correlation between in-vivo AOP and TFC.

### Multivariate analysis

Linear discriminant analysis of the data for in-vitro AOP, in-vivo AOP, TPC and TFC in the four chicory cultivars was performed to model the differences among the classes of data regarding the fertiliser treatments. Among the measured variables, TPC shows the highest variation, and in-vivo AOP the lowest. The distribution of chicory samples on the three fertiliser treatments and the Control was 100% successful. Samples of ‘Verona’ chicory with the mineral fertiliser treatment were classified into the Control group, and the ‘Castelfranco’ chicory samples from the Control group were classified into the group of chicories with the mineral fertiliser treatment.

## Conclusions

This study shows that the combination of an organic and a mineral fertiliser provided the highest in-vitro AOPs for the red chicory cultivars, and the highest in-vivo AOPs for the red and red-spotted chicory cultivars. Furthermore, the fertiliser treatments had different impacts on the red and red-spotted chicory cultivars in terms of TFC and TPC, which resulted in different AOPs in-vitro and in-vivo. Among the cultivars examined, ‘Castelfranco’ with the Organic+Mineral fertiliser treatment showed the highest AOP in vivo, with the lowest TPC and TFC. These data show that the in-vitro AOP is well reflected in the in-vivo AOP for the red chicory cultivars, but less so for the red-spotted ‘Castelfranco’ cultivar. The expected negative relations were seen between the in-vitro and in-vivo AOPs for the red cultivars, while a positive relation was seen for the red-spotted cultivar. As the in-vivo AOP reflects the cellular physiology, these data are more useful compared to the in-vitro AOP results. With the linear discriminant analysis model, 100% correct classification was predicted for samples of chicory for the combination organic plus mineral fertiliser treatment. Thus, based on these data for the in-vivo AOP for the red and red-spotted chicory cultivars, the Organic+Mineral fertiliser treatment can be recommended.

## Methods

### Plant materials and sample preparation

Four commercial cultivars of chicory (*Chicorium intybus* L.) were included: three red cultivars ‘Treviso’ (Semenarna Ljubljana, Slovenia), ‘Verona’ (Semenarna Ljubljana, Slovenia) and ‘Anivip’ (L’Ortolano, Italy), and one red-spotted cultivar ‘Castelfranco’ (Semenarna Ljubljana, Slovenia). The seeds were purchased from commercial seed companies mentioned above. Selected cultivars of chicory were inscribed in the Slovene list of varieties under register numbers: CCI003 ‘Anivip’ [34], CCI007 ‘Castelfranco’, CCI025 ‘Treviso’ and CCI027 ‘Verona’ [35]. All definitive standard samples of the chicory cultivars are stored according to the National legislation in the official public seed repository at Agricultural institute of Slovenia, Ljubljana, under the following numbers: 0513/2017SV ‘Anivip’, 0000/2007MSV ‘Castelfranco’, 0159/2008MSV ‘Treviso’ and 0518/2008MSV ‘Verona’. Cultivar ‘Treviso’ is the classic tall chicory with upright red and white striped leaves and large pure white stems. Chicory cultivar ‘Verona’ forms round medium sized heads with dark wine red colour leaves and prominent white veins. Cultivar ‘Castelfranco’ forms big round heads with apple green flecked leaves with wine red inner. Chicory ‘Anivip’ is an autochthonous Slovene cultivar which forms round large heads with wine red coloured leaves.

This experiment was carried out in the first half of 2012 in a glasshouse at the Biotechnical Faculty of the University of Ljubljana, Ljubljana, Slovenia (46° 04′ N, 14° 31′ W; 320 m. a.s.l.). Along with the control (no added fertilisers), different types of fertilisers were tested: an organic fertiliser, a mineral fertiliser, and a combination of an organic and a mineral fertiliser. In each of these four cultivars, the same four treatments were applied in a completely randomised factorial design, as: no added fertiliser (Control); addition of the organic fertiliser Plantella Organic (Organic; N-P-K 3–3-2; 67.5 g/pot; Unichem, Slovenia); addition of the mineral fertiliser ENTEC perfect (Mineral; N-P-K 14–7-17; 7.9 g/pot; EuroChem Agro, Germany); and addition of the combination of the organic fertiliser Plantella Organic (2.5 g/pot) and the water-soluble mineral fertiliser Kristalon Blue (Organic+Mineral; N-P-K 19–6-20; applied after 1 month, during watering once per week with 3.5 g/L; Yara, UK). Sowing time and method, plant care, water-soluble fertilizer application during the growth and harvesting time and process was carried out as previously described by Sinkovič et al. [14]. Briefly, plastic pots filled with virgin soil and/or added fertilisers were placed on rolling benches in a heated glasshouse compartment and watered as needed.

For the preparation of samples only undamaged uniform leaves were collected. For each sample, 10 g fresh leaves were chopped up using a ceramic knife, and extracted in a plastic vial with 10 g 100% methanol. The tissue in the methanol was then homogenised using a laboratory mixer (20,500 rpm for 5 min; Ultraturax T 25). The samples were stored at − 20 °C until analysed.

To determine the dry matter content (%DM), leaves from all of the plants were dried in a laboratory oven at 80 °C for 28 h. The dry matter content ranged from 6.8 to 14.8% of the leaf fresh weight (FW).

### Reagents and chemicals

The water used for sample extraction and analysis was from a Milli-Q water purification system (Millipore, Billerica, MA, USA). Methanol, Folin–Ciocalteu reagent, the standards of Trolox, gallic acid and quercetin, and 2,2-diphenyl-1-picrylhydrazyl radical (DPPH) were from Merck (Darmstadt, Germany). 2′,7′-Dichlorofluorescin diacetate (H_2_DCFDA) was from Sigma-Aldrich (St. Louis, USA).

### *Determination of* in-vitro *antioxidant potential*

The in-vitro AOP was determined using the DPPH free radical scavenging assay [22]. For the reference value, 120 μL methanol and 1.5 mL DPPH solution (4 mg/20 mL methanol) were mixed in a microcentrifuge tube (Eppendorf, Germany), with the samples run in triplicate. For the chicory extracts, 120 μL extraction solution was mixed with 1.5 mL DPPH solution, also in microcentrifuge tubes and in triplicate. For the blank, 120 μL of the extraction solution was mixed with 1.5 mL methanol. After 15 min at room temperature, the absorbance was measured at 517 nm using a spectrophotometer (MRX; Dynex Technologies). The AOP is expressed as Trolox equivalents (g TE/kg FW) using a calibration curve that ranged from 40 mg TE/L to 220 mg TE/L (R^2^ = 0.9900).

### *Determination of* in-vivo *antioxidant potential*

For determination of the in-vivo AOP, 2 mL defrosted leaf homogenate was centrifuged (14,000×*g*, 5 min) and the supernatant was filtered (pore size, 0.2 μm). The in-vivo AOP was determined in the yeast *Saccharomyces cerevisiae* from the Culture Collection of Industrial Microorganisms (University of Ljubljana, Biotechnical Faculty, Ljubljana, Slovenia) by measuring intracellular oxidation. The cultivation of *S. cerevisiae* was carried out in YPD medium (10 g/L of yeast extract, Biolife; 20 g/L of peptone, Biolife; 20 g/L of glucose, Merck) at 28 °C and 220 rpm. In the stationary phase the yeast cells were centrifuged (4000×*g*, 3 min), washed once and resuspended in phosphate-buffered saline to reach 1 × 10^8^ cell/mL. The *S. cerevisiae* were further incubated at 28 °C for 96 h, with agitation at 220 rpm. They were then treated with the chicory methanol extracts at 1% (v/v) for 2 h at 28 °C, with agitation at 220 rpm. These treatments were sampled for determination of *S. cerevisiae* intracellular oxidation.

The *S. cerevisiae* intracellular oxidation was determined using 2`,7`-dichlorofluorescein (H_2_DCF), which reacts with oxidants. This was added as H_2_DCFDA, which penetrates the plasma membrane and is hydrolysed inside the cells by non-specific esterases. The non-fluorescent H_2_DCF that is produced can then be oxidised to the fluorescent 2`,7`-dichlorofluorescin (DCF), the levels of which were determined fluorimetrically [36].

For this assay, the *S. cerevisiae* cells from 2-mL cell cultures were sedimented by centrifugation (14,000×*g*, 5 min), and washed three times with 50 mM potassium phosphate buffer (pH 7.8). The cell pellets were then resuspended in 50 mM potassium phosphate buffer at 10% (v/v), and preincubated at 28 °C for 5 min. The reactive oxygen species sensing dye H_2_DCFDA was added from a 1 mM stock solution in methanol, to a final concentration of 10 μM. After incubation for 20 min at 28 °C with agitation at 220 rpm, with the fluorescence of the cell suspensions was measured using a microplate reader (Safire II; Tecan). The excitation and emission wavelengths of DCF were 488 nm and 520 nm [37]. The data are expressed as proportions of fluorescence relative to control (untreated cells), and thus lower values indicate higher in-vivo AOP, and vice versa.

### Determination of total phenolics content

The TPC was determined using a spectrophotometer and following the Folin–Ciocalteu method, as first described by Singleton and Rossi [38], and as slightly modified. The samples were centrifuged at 13.2 relative centrifugal force for 5 min (5415 D centrifuge; Eppendorf) and the supernatants were diluted with deionised water at a ratio of 1:1 (v/v) or 2:1 (v/v). Gallic acid solutions were used for the construction of the calibration curve. Briefly, 1 mL of each methanol fraction was mixed with 120 mL deionised water and 5 mL diluted (1:17) Folin–Ciocalteu reagent, in a 100-mL flask. The solutions were mixed well, and after 30 s and before 8 min, 15 mL of a solution of 20% (w/v) Na_2_CO_3_ was added. After an incubation for 2 h at 20 °C [39], absorption was measured at 765 nm. The seven-point calibration curve ranged from 3 mg/L to 150 mg/L of gallic acid (R2 = 0.9998). Data were expressed as gallic acid equivalents (mg GAE/100 g FW).

### Determination of total flavonoids content

The TFC was measured according to a method described previously [40]. Before the analysis, the samples were centrifuged at 13.2 relative centrifugal force for 5 min. The supernatant (250 μL) was added to 750 μL 95% (v/v) ethanol, 50 μL 10% (w/v) aluminium chloride hexahydrate, 50 μL 1 M potassium acetate, and 1.4 mL deionised water, and mixed. After incubation at room temperature for 40 min, the absorbance of the reaction mixture was measured at 415 nm, against a blank of deionised water. Quercetin was used as the standard. A seven-point standard curve was constructed, which ranged from 0.3 mg QE/100 mL to 15 mg QE/100 mL (R^2^ = 0.9981). The data are expressed as mg QE/100 g FW.

### Statistical analysis

The experimental data were evaluated statistically using SPSS, version 15.0 for Windows, as the evaluation version (SPSS Inc., Chicago, IL, USA). Descriptive statistics were calculated for each chicory cultivar and fertiliser treatment. The data were tested for normal distribution; and the main effects of fertiliser treatment (Control, Organic, Mineral, Organic+Mineral), cultivar (‘Trevisio’, ‘Verona’, ‘Anivip’, ‘Castelfranco’) and fertiliser treatment × cultivar, which were tested using the general linear model procedure. Means were calculated for the experimental groups using the least-squared means procedure, and were compared at the 5% probability level. Here, the interaction of fertiliser treatment × cultivar did not have any effects on the data, while each factor did have effects individually (i.e., fertiliser, cultivar). The relationships between the parameters observed were examined according to Pearson’s correlation coefficients. Linear discriminant analysis of the data was performed for in-vitro AOP, in-vivo AOP, TPC and TFC, to model the differences among the classes of data regarding the fertiliser treatments. A biplot was constructed for the first two functions, to illustrate how the treated cultivars varied and to define which parameter defined the greatest variation.

## Supplementary information

**Additional file 1: Table S1.** Influence of the fertiliser treatments on the in-vitro and in-vivo antioxidant potential (AOP) for the four chicory cultivars.

**Additional file 2: Table S2.** Influence of the fertiliser treatments on the total phenolics content for the four chicory cultivars, as determined by the Folin-Ciocalteu method.

**Additional file 3: Table S3.** Influence of fertiliser treatments on the total flavonoids content for the four chicory cultivars.

## Data Availability

The datasets used and/or analysed during the current study available from the corresponding author on reasonable request.

## Abbreviations

AO: Antioxidant potential
DM: Dry matter
DPPH: 2,2-diphenyl-1-picrylhydrazyl radical
FW: Fresh weight
GAE: Gallic acid equivalents
QE: Quercetin equivalents
TE: Trolox equivalents
TFC: Total flavonoids content
TPC: Total phenolics content
