# Supplementary material for: In-vitro and in-vivo antioxidant assays of chicory plants (Cichorium intybus L.) as influenced by organic and conventional fertilisers
Source: BMC Plant Biol. 2020 Jan 20;20:36. doi: 10.1186/s12870-020-2256-2 (PMC6972005; doi:10.1186/s12870-020-2256-2)
Supplement: Supplementary file 1 — Additional file 1: Table S1. Influence of the fertiliser treatments on the in-vitro and in-vivo antioxidant potential (AOP) for the four chicory cultivars. [file 12870_2020_2256_MOESM1_ESM.docx]

**Table S1.** Influence of the fertiliser treatments on the *in-vitro* and *in-vivo* antioxidant potential (AOP) for the four chicory cultivars.

| **Chicory** | **AOP according to fertiliser use** | | | | | | | |
| --- | --- | --- | --- | --- | --- | --- | --- | --- |
| **cultivar** | ***In vitro* (g TE/kg FW)** | | | | ***In vivo* (relative fluorescence)** | | | |
|  | **Control** | **Organic** | **Mineral** | **Organic+ Mineral** | **Control** | **Organic** | **Mineral** | **Organic+ Mineral** |
| ‘Trevisio’ | 0.55 ±0.01 ^cB^ | 0.85 ±0.01 ^bA^ | 0.45 ±0.00 ^dA^ | 1.06 ±0.02 ^aC^ | 0.98 ±0.03 ^aA^ | 0.94 ±0.04 ^aA^ | 0.74 ±0.02 ^bC^ | 0.71 ±0.02 ^bB^ |
| ‘Verona’ | 0.92 ±0.01 ^bA^ | 0.65 ±0.01 ^cB^ | 0.42 ±0.00 ^dB^ | 1.11 ±0.02 ^aB^ | 0.95 ±0.02 ^aAB^ | 0.97 ±0.01 ^aA^ | 0.95 ±0.03 ^aA^ | 0.81 ±0.04 ^bA^ |
| ‘Anivip’ | 0.41 ±0.01 ^bC^ | 0.28 ±0.00 ^cD^ | 0.42 ±0.00 ^bB^ | 1.57 ±0.01 ^aA^ | 0.90 ±0.03 ^aBC^ | 0.77 ±0.02 ^bB^ | 0.80 ±0.05 ^abBC^ | 0.72 ±0.05 ^bB^ |
| ‘Castelfranco’ | 0.31 ±0.02 ^bD^ | 0.38 ±0.01 ^aC^ | 0.30 ±0.00 ^bC^ | 0.26 ±0.00 ^cD^ | 0.80 ±0.01 ^aC^ | 0.61 ±0.01 ^cC^ | 0.66 ±0.01 ^bD^ | 0.56 ±0.01 ^dC^ |

TE, Trolox equivalents; FW, fresh weight

Data are means ±standard deviation (n = 3)

Means with different superscript small letters (a, b, c, d) along a row within the *in-vivo* AOP and *in-vitro* AOP data columns separately are significantly different (*P* <0.05; i.e., differences between fertilizer treatments within the AOP classifications)

Means with different superscript capital letters (A, B, C, D) down the data columns are significantly different (*P* <0.05; i.e., differences between the cultivars within the AOP classifications)
