# Supplementary material for: In-vitro and in-vivo antioxidant assays of chicory plants (Cichorium intybus L.) as influenced by organic and conventional fertilisers
Source: BMC Plant Biol. 2020 Jan 20;20:36. doi: 10.1186/s12870-020-2256-2 (PMC6972005; doi:10.1186/s12870-020-2256-2)
Supplement: Supplementary file 2 — Additional file 2: Table S2. Influence of the fertiliser treatments on the total phenolics content for the four chicory cultivars, as determined by the Folin-Ciocalteu method. [file 12870_2020_2256_MOESM2_ESM.docx]

**Table S2.** Influence of the fertiliser treatments on the total phenolics content for the four chicory cultivars, as determined by the Folin-Ciocalteu method.

| **Chicory** | **Total phenolics content (mg GAE/100 g FW) according to fertiliser use** | | | |
| --- | --- | --- | --- | --- |
| **cultivar** | **Control** | **Organic** | **Mineral** | **Organic+Mineral** |
| ‘Trevisio’ | 67.57 ±0.95 ^cB^ | 71.23 ±1.65 ^bA^ | 37.53 ±0.40 ^dD^ | 94.33 ±0.42 ^aC^ |
| ‘Verona’ | 114.93 ±3.10 ^aA^ | 59.40 ±1.11 ^cB^ | 61.20 ±1.13 ^cA^ | 100.80 ±2.55 ^bB^ |
| ‘Anivip’ | 49.53 ±1.19 ^bC^ | 40.57 ±0.75 ^cD^ | 41.60 ±1.30 ^cC^ | 120.20 ±3.17 ^aA^ |
| ‘Castelfranco’ | 39.27 ±0.47 ^cD^ | 59.53 ±0.93 ^aB^ | 49.70 ±1.08 ^bB^ | 28.27 ±0.06 ^dD^ |

GAE, gallic acid equivalents; FW, fresh weight

Data are means ±standard deviation (n = 3)

Means with different superscript small letters (a, b, c, d) along a row are significantly different (*P* <0.05; i.e., differences between the fertiliser treatments)

Means with different superscript capital letters (A, B, C, D) down a column are significantly different (*P* <0.05; i.e., differences between the cultivars)
