# Supplementary material for: In-vitro and in-vivo antioxidant assays of chicory plants (Cichorium intybus L.) as influenced by organic and conventional fertilisers
Source: BMC Plant Biol. 2020 Jan 20;20:36. doi: 10.1186/s12870-020-2256-2 (PMC6972005; doi:10.1186/s12870-020-2256-2)
Supplement: Supplementary file 3 — Additional file 3: Table S3. Influence of fertiliser treatments on the total flavonoids content for the four chicory cultivars. [file 12870_2020_2256_MOESM3_ESM.docx]

**Table S3.** Influence of fertiliser treatments on the total flavonoids content for the four chicory cultivars.

| **Chicory** | **Total flavonoids content (mg QE/100 g FW) according to fertiliser use** | | | |
| --- | --- | --- | --- | --- |
| **cultivar** | **Control** | **Organic** | **Mineral** | **Organic+Mineral** |
| ‘Trevisio’ | 3.64 ±0.41 ^bB^ | 2.13 ±0.13 ^cA^ | 2.15 ±0.12 ^cBC^ | 9.92 ±0.48 ^aB^ |
| ‘Verona’ | 9.22 ±0.10 ^bA^ | 2.29 ±0.16 ^dA^ | 6.83 ±0.31 ^cA^ | 12.58 ±0.86 ^aA^ |
| ‘Anivip’ | 1.89 ±0.26 ^cC^ | 1.28 ±0.05 ^cB^ | 2.67 ±0.29 ^bB^ | 4.39 ±0.39 ^aC^ |
| ‘Castelfranco’ | 1.90 ±0.25 ^aC^ | 0.94 ±0.10 ^cC^ | 2.10 ±0.14 ^aBC^ | 1.25 ±0.07 ^bD^ |

QE, quercetin equivalents; FW, fresh weight

Data are means ±standard deviation (n = 3)

Means with different superscript small letters (a, b, c, d) along a row are significantly different (*P* <0.05; i.e., differences between the fertiliser treatments)

Means with different superscript capital letters (A, B, C, D) down a column are significantly different (*P* <0.05; i.e., differences between the cultivars).
